# Visualization of basilar artery atherosclerotic plaques by conventional T2-weighted magnetic resonance imaging: A case-control study

**DOI:** 10.1371/journal.pone.0212570

**Published:** 2019-02-26

**Authors:** Mi Ji Lee, Soohyun Cho, Jihoon Cha, Seonwoo Kim, Sung Tae Kim, Oh Young Bang, Chin-Sang Chung, Kwang Ho Lee, Gyeong-Moon Kim

**Affiliations:** 1 Department of Neurology, Samsung Medical Center, Sungkyunkwan University School of Medicine, Seoul, Korea; 2 Department of Radiology, Samsung Medical Center, Sungkyunkwan University School of Medicine, Seoul, Korea; 3 Biostatistics Team, Samsung Biomedical Research Institute, Samsung Medical Center, Seoul, Korea; Universitatsklinikum Freiburg, GERMANY

## Abstract

**Objective:**

In vivo visualization of intracranial atherosclerotic plaque has been performed only with high-resolution magnetic resonance imaging (HRMR). We investigated whether atherosclerotic plaque of the basilar artery (BA) can be identified in conventional magnetic resonance imaging (MRI).

**Methods:**

Patients with acute ischemic stroke who had BA stenosis (“symptomatic BAA”) were retrospectively recruited using the prospective stroke registry. In the HRMR databank, subjects without BA stenosis were recruited and classified as those with silent plaque (“silent BAA”) and without any plaque (“normal controls”). Outer diameter of the BA and T2 plaque sign (an eccentric or complete obscuration of normal flow-void) within the BA were assessed by two blinded raters using conventional T2 MRI.

**Results:**

Seventy-five patients with symptomatic BAA, 40 with asymptomatic BAA, and 36 normal controls were included in the study. Maximal BA diameter was significantly larger in symptomatic BAA patients with <30%, 30–50%, 50–70%, and >70% stenosis (all p<0.01 in each subgroup) and silent BAA subjects (p = 0.018) than controls. T2 plaque signs were present in 46 (61.3%) patients with symptomatic BAA and 6 (14.6%) subjects with asymptomatic BAA, while none in normal controls (p <0.001 and 0.057, respectively). Detection rates were increased with an increase in stenosis degree (25.0% in <30% stenosis, 57.9% in 30–50% stenosis, 38.5% in 50–70% stenosis, 92.3% in 70–99% stenosis, and 100.0% in occlusion).

**Conclusions:**

Our data suggest that BA atherosclerosis can be detected by conventional MRI. When the use of HRMR is limited, conventional MR imaging may give additive information to clinicians.

## Introduction

Intracranial large artery atherosclerosis is one of the major causes of ischemic stroke [1]. Mechanisms of stroke caused by intracranial atherosclerosis include artery-to-artery embolism [2], branch occlusive disease [3], in-situ thrombo-occlusion, and less commonly, hemodynamic ischemia [4]. Although it is the atherosclerotic plaque that plays a causative role, only the degree of stenosis has been focused on the diagnosis of large artery atherosclerotic stroke in modern classification systems of stroke mechanism.

Through advances in imaging technology, it is possible to visualize atherosclerotic plaques in intracranial arteries with high-resolution MR imaging (HRMR) [5]. Recent researches based on HRMR allowed us to understand features of intracranial plaques such as arterial remodelling, eccentric plaques, plaque enhancement, and intraplaque hemorrhage [6,7]. However, despite its several advantages, the use of HRMR is often limited because of its cost and availability. Additional scanning time is another limitation, especially in patients with unstable medical conditions.

Conventional MR imaging is the preferred method to diagnose ischemic stroke in most centers [8]. Conventional MRI has been considered to have a limited ability to show atherosclerotic plaque due to relatively large slice thickness and in-plane voxel size. Incomplete suppression of intraluminal flow-related signal is another limitation, which can mimic a plaque [9]. However, the flow direction of the BA is perpendicular to the axial plane and consequently less vulnerable to plaque-mimicking artifacts. As a result, disappearance of the flow void signals of the BA implicates an occlusion [10–12]. In this context, eccentric loss of flow void signals may be a sign of the presence of atherosclerotic plaque. Although less sensitive than HRMR, the presence of atherosclerotic plaque has been documented by conventional T2 axial imaging in a previous case study [13]. Moreover, enlarged BA signal in T2 imaging may indicate positive remodelling, which is considered to occur earlier in the development of atherosclerosis than luminal stenosis [14]. However, there is no study that assessed the sensitivity and specificity of conventional MR imaging in diagnosis of BA atherosclerosis.

The aim of this study was to determine the diagnostic value of the BA diameter and eccentric loss of flow void, as measured by T2-weighted axial imaging, for the BA atherosclerosis (BAA) with different degrees.

## Materials and methods

### Patients

Using a prospectively maintained stroke registry, patients with acute ischemic stroke in the posterior circulation between March 2011 and June 2015 were retrospectively identified. Patients whose stroke mechanism was classified as BAA of any degree were included for the study (“symptomatic BAA” group). We excluded patients who had high-risk of a cardioembolic source, those with vertebral or BA dissection, those with vertebrobasilar dolichoectasia, and those without pretreatment T2-weighted MR imaging (T2WI).

### Silent BAA group and normal controls

Among subjects who underwent HRMR for reasons other than BA atherosclerosis, those within the same age range as the patient group were retrospectively identified. After a review of their conventional MR angiogram, 111 subjects without BA stenosis were selected. Two trained investigators (M.J.L. with 9 years of experience in neurology and J.C. with 13 years of experience in neuroradiology) reviewed HRMR data and excluded 35 subjects for the following reasons: incomplete coverage of the BA (n = 12), unavailable conventional T2WI (n = 9), images taken using a different protocol (n = 13), and poor quality due to excessive patient motion (n = 1). From the remaining 76 subjects, 40 were judged to have atherosclerotic wall thickening or plaques and assigned to the silent BAA group. The remaining 36 subjects who showed completely normal BA wall served as normal controls.

### Evaluations

Patients were evaluated based on demographic characteristics, medical history, vascular risk factors, routine blood tests, brain imaging, and cardiological assessments. Stroke mechanisms were classified using the causative classification of stroke (CCS) system [15] and determined by consensus of two stroke neurologists (M.J.L. with 9 years of experience in neurology and G-M.K. with 28 years of experience in neurology). Based on the CCS system, patients with >50% steno-occlusive lesions within the BA (“Large artery atherosclerosis (LAA)” category) and those with both an isolated pontine infarction >20mm in diameter and irregularities or mild stenosis within the relevant segment of the BA (“Branch atheromatous disease (BAD)” category) were classified as symptomatic BAA. The Samsung Medical Center institutional review board approved this study.

### Conventional MR protocols and image analysis

All patients and controls underwent conventional MR imaging using a 3T MR scanner (Achieva, Philips Medical Systems, Best, Netherlands). In the patient group, MR imaging was performed at median 1 (IQR 0–3, range 0–9) days after stroke onset. T2WI and T1WI were performed using a three-dimensional spin echo sequence with the following parameters respectively: T2WI, repetition time (TR)/echo time (TE) = 3000 / 80 ms, field of view (FOV) = 240 mm, acquisition matrix = 320 × 249, voxel size = 0.469 × 0.469 mm^2^, echo train length = 15, number of signal averages = 1, slice thickness = 5 mm, interslice gap = 1.5 mm, scanning time = 120 s; T1WI, TR/TE = 500 / 10 ms, FOV = 240 mm, voxel size = 0.469 × 0.469 mm^2^, echo train length = 1, number of signal averages = 1, slice thickness = 5 mm, interslice gap = 1.5 mm, scanning time = 150 s. Time-of-flight MRA of the intracranial arteries was also obtained with following parameters: TR / TE 25 / 3.5 ms, FOV = 170 mm, acquisition matrix = 880 × 450, slice thickness = 0.45 mm, 80 slices over contiguous sampling, 20° flip angle.

**Fig 1:**
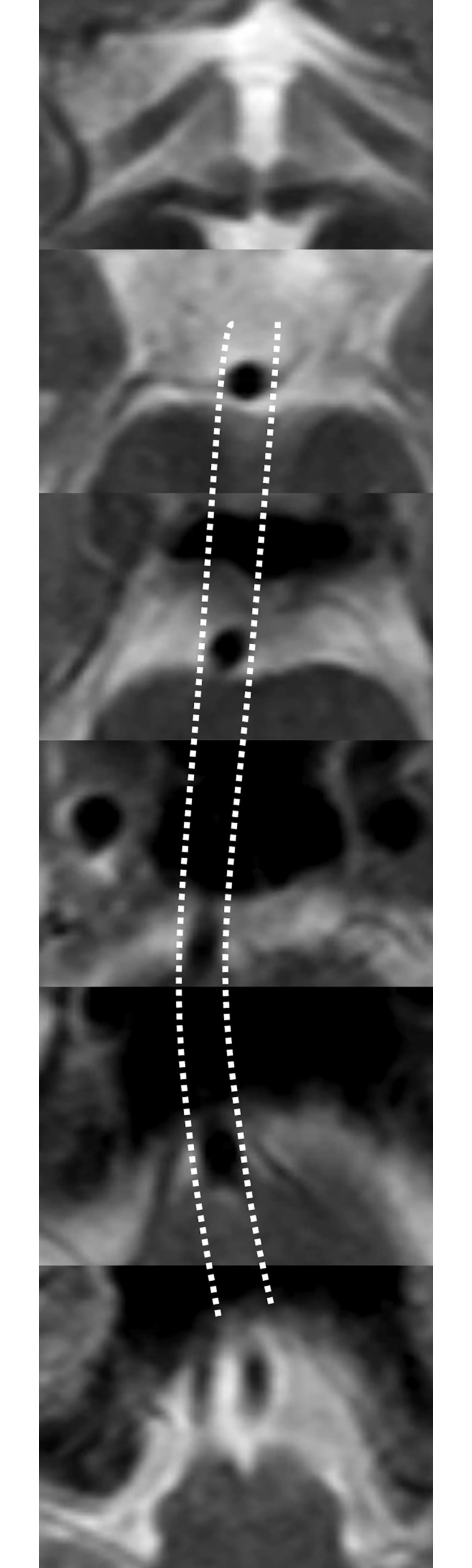
Determination of the course of the BA.

**Fig 2:**
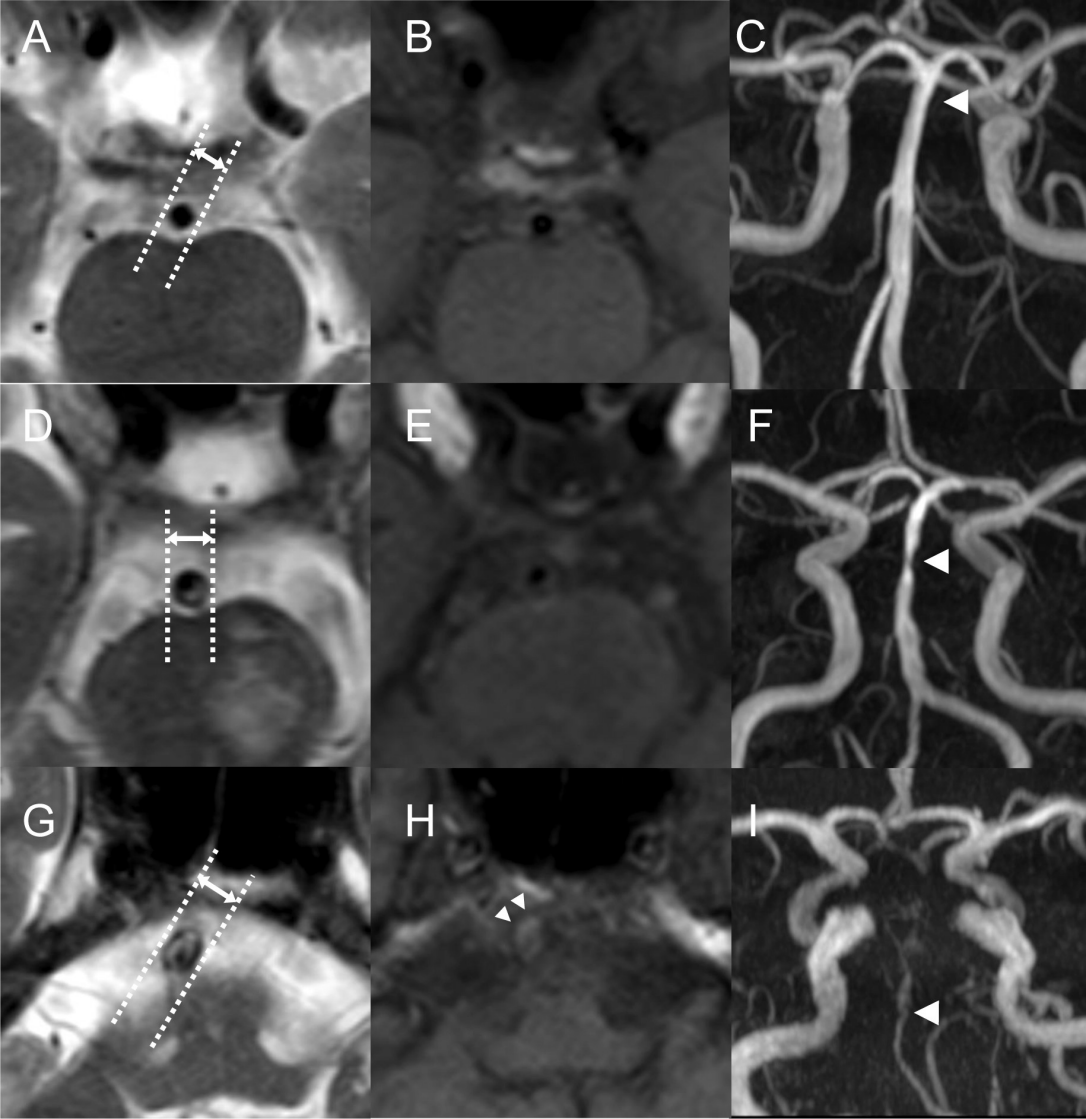
Representative basilar artery images. Left column, T2-weighted imaging; middle column, T1-weighted imaging; right column, MR angiogram. (A-C) Normal findings, with a diameter of 2.65 mm. (D-F) Eccentric T2 plaque sign (*) and increased diameter (4.51mm) were noted in a patient with 50%-69% stenosis. (G-I) T2 plaque sign (*), an increased diameter (5.4mm), and T1 hyperintensity within the plaque (H) in a patient with severe steno-occlusion of the basilar artery.

T2-weighted axial images were reviewed by two investigators (M.J.L. and J.C.) blinded to patient information. To measure BA outer diameter, 2–4 axial slices showing the BA were reviewed to determine the course of the BA (Fig 1). The outer diameter of the BA was measured perpendicular to the course of the BA. The axial cut showing BA segment of the maximal diameter was used for the measurement. The outer diameter of BA was used for the analysis (Fig 2). Because the axis of flow direction might change within the single axial cut in cases with severe tortuosity, the cut showing the most straight course whose distal and proximal cuts have the same axis of flow direction was selected to measure maximal diameter. Stenosis degree was graded as 0, normal (normal flow signal); 1, 30% (focal indentation but > 50% of lumen visualized); 2, 50% (>50% reduction of flow signal but no flow gap); 3, 70% (flow gap but visible flow signals distal to the stenotic segment); and 4, occlusion (no distal flow signal visualized). This grading system was validated in our previous study [16].

T2 plaque sign was defined as a clearly marginated eccentric lesion within the BA, whose signal intensity was higher than the normal flow-voids [13]. Typical cases are illustrated in Fig 2. Because of flow diversion, measurement at the most proximal and distal segments of the BA were avoided. Once the plaque is identified, the location of plaque was classified as dorsal, ventral, lateral, and the combination. The diameter of plaque (“plaque diameter”) was determined with the length between the inner lumen and outer wall in the middle of plaque. T1 hyperintensity was determined if T1 signal along the BA wall was higher than adjacent muscles (Fig 2, S1 Fig). Intraluminal T1 hyperintensity, which might be related to slow flow [17], was not considered significant (S1 Fig). Intra-observer reliability for maximal BA diameter was good [intraclass correlation coefficient (ICC) = 0.913] and kappa values for T2 plaque sign and T1 hyperintensity were 0.761 and 0.896.

### HRMR protocols

In addition to conventional MRI, HRMR data were available in 20 patients with symptomatic BAA patients, all subjects with silent BAA subjects, and normal controls. HRMR were performed using the same 3.0T MRI scanner (Ingenia, Philips Medical Systems, Best, The Netherlands) with a 20-channel sensitivity-encoding (SENSE) head coil. Three-dimensional (3D) time-of-flight MR angiography [TR/TE = 25 / 3.5 ms; flip angle (FA) = 20°; matrix = 880 × 332; slice thickness = 0.6 mm; FOV = 250 mm; acquisition time, 4 min 53 s], proton density volumetric isotropic TSE acquisition (VISTA): [TR/TE = 2000/36 ms; turbo spin echo (TSE) factor = 63; SENSE factor = 2; number of signal average (NSA) = 1; matrix = 360 × 360; FOV = 180 mm; 0.5 mm isotropic voxel; acquisition time, 8 min 4 s], and pre- and post-contrast T1 VISTA: [TR/TE = 350/20 ms; TSE factor = 25; SENSE factor = 2; NSA = 3; matrix = 256 × 256; FOV = 180 mm; 0.7 mm isotropic voxel; acquisition time, 7 min] images were obtained. T1 VISTA images were obtained with improved motion-sensitized driven-equilibrium preparation for blood suppression (velocity encoding = 3 cm/s). Post-contrast T1 VISTA images were acquired after intravenous injection of contrast agent [Dotarem (Gadoterate Meglumine); Guerbet, Aulnay-sous-Bois, France; 0.1 mmol/kg body weight]. Two investigators (M.J.L. and J.C.) reviewed the HRMR data for the outer diameter of the BA and presence of atherosclerotic plaques. Proton-density VISTA imaging was used for the measurement of outer wall diameter. The presence and maximal diameter of atherosclerotic plaque were identified by using pre- and post-contrast T1 VISTA and proton-density VISTA imaging.

### Statistical analysis

All data are presented as means (standard deviation) or frequencies (percentages), unless otherwise specified. The Student’s t-test or Mann–Whitney U test was used to compare continuous variables and a Pearson's chi-square test or Fisher's exact test was used to compare categorical variables between groups. Analysis of covariance (ANCOVA) was performed to adjust age effect to the group assignment.

Sensitivity and specificity were calculated for the presence of BAA of any degree and stenosis of >30%, >50% and >70%. Receiver operating characteristic (ROC) curves were used to evaluate the predictive performance and the optimal cut-off value of maximal diameter for differentiating patients from controls. For the validation analysis, the Spearman’s correlation analysis was performed to test the correlation between the conventional T2WI-based and HRMR-based measurements. For variables which shows a good correlation, the linear regression analysis was performed to test the explanatory power of the conventional T2WI-based measurement on the HRMR-based measurement. A regression equation was constructed to enable the conversion of T2WI-based measurement to HRMR-based measurement and to reflect the difference of two variables (a constant). In this analysis, the HRMR-based BA diameter was log-transformed to achieve a normal distribution. Statistical analyses were performed using SPSS version 18.0 software (SPSS Inc., Chicago, IL, USA). A p-value <0.05 was considered statistically significant.

## Results

### Demographics

Among the 81 patients with symptomatic BAA identified, 6 were excluded (Lack of pretreatment conventional T2WI, N = 4; and BA dissection, N = 2). Seventy-five patients with symptomatic BAA, 40 with asymptomatic BAA, and 36 normal controls were finally included in the study. Demographics of study subjects are shown in Table 1. Patients with BAA were older and had more comorbid risk factors including hypertension, diabetes, dyslipidemia, and current tobacco use than controls. The presence of BA hypoplasia and fetal circulation were not different between groups (Table 1).

**Table 1:** Characteristics of study subjects.

	Normal controls (n = 36)	Silent BAA (n = 40)	Symptomatic BAA (n = 75)	*P*
Age, y, mean (range)	58.4 (51–81)	63.5 (51–81)	69.2 (51–81)	<0.001
Male sex	17 (47.2%)	16 (40.0%)	47 (62.7%)	0.064
Hypertension	16 (44.4%)	24 (60.0%)	58 (77.3%)	0.002
Diabetes	5 (13.9%)	9 (25.0%)	44 (58.7%)	<0.001
Dyslipidemia	14 (38.9%)	14 (35.0%)	44 (59.5%)	0.034
Coronary artery disease	4 (11.1%)	4 (10.0%)	15 (20.0%)	0.370
Atrial fibrillation	0 (0.0%)	2 (5.0%)	0 (0.0%)	0.126
Peripheral arterial occlusive disease	0 (0.0%)	1 (2.5%)	0 (0.0%)	0.507
Smoking	3 (8.3%)	5 (12.5%)	18 (24.0%)	0.076
Previous Stroke/TIA	5 (13.9%)	2 (5.0%)	20 (26.7%)	0.011
Basilar artery stenosis by MRA				<0.001
None	36 (100.0%)	40 (100.0%)	-	
<30%	-	-	16 (21.3%)	
30–50%	-	-	19 (25.3%)	
50–70%	-	-	13 (17.3%)	
70–99%	-	-	13 (17.3%)	
Occlusion	-	-	14 (18.7%)	
Hypoplasia	1 (2.8%)	2 (4.9%)	3 (4.0%)	>0.999
Fetal circulation	1 (2.8%)	2 (4.9%)	2 (2.7%)	0.842
DWI lesion				NA
Isolated pontine infarct	-	-	49 (65.3%)	
PCA, SCA, or AICA	-	-	11 (14.7%)	
Both	-	-	6 (8.0%)	
Negative	-	-	9 (12.0%)	
Stroke mechanism				NA
LAA			40 (53.3%)	
BAD			35 (46.7%)	

Abbreviation: BAA = basilar artery atherosclerosis; TIA = transient ischemic attack; DWI = diffusion-weighted imaging; LAA = large-artery atherosclerosis; BAD = branch atheromatous disease.

### Conventional MR markers in patients and controls

The results of conventional MRI analysis are summarized in Table 2. The mean maximal diameter of the BA was 2.7 (0.45) mm in normal controls, 3.0 (0.71) mm in subjects with silent BAA (p = 0.018 vs normal controls), and 3.9 (0.84) mm in patients with symptomatic BAA (p<0.001 vs normal controls). Subgroups of each degree of stenosis showed significantly larger BA diameters than those of controls (Table 2). The BA maximal diameter was not correlated with age (rho = -0.098, p = 0.571).

**Table 2:** Conventional MR markers in patients with basilar artery atherosclerosis (BAA) and normal controls.

	Stenosis degree	Outer wall diameter (mm)	p	Plaque diameter (mm)	T2 plaque sign	p	T1 hyperintensity*	p	T2 plaque or T1 hyperintensity	p
Normal Controls (n = 36)		2.7 (0.45)		NA	0 (0.0%)		0 (0.0%)		0 (0.0%)	
Asymptomatic BAA (n = 40)	<30%	3.0 (0.71)	0.018	1.0 (0.62)	5 (12.2%)	0.057	5 (12.2%)	0.057	6 (16.7%)	0.012
Symptomatic BAA (n = 75)	all (n = 75)	3.9 (0.84)	<0.001	1.91 (1.04)	46 (61.3%)	<0.001	25 (33.8%)	<0.001	50 (66.7%)	<0.001
BAD	<30%(n = 16)	3.7 (0.83)	<0.001	1.0 (0.62)	4 (25.0%)	0.007	3 (18.8%)	0.029	6 (37.5%)	<0.001
	30–50% (n = 19)	3.8 (0.77)	<0.001	1.3 (0.60)	11 (57.9%)	<0.001	5 (26.3%)	0.004	12 (63.2%)	<0.001
LAA	50–70% (n = 13)	4.4 (0.84)	<0.001	1.8 (0.31)	5 (38.5%)	0.001	1 (7.7%)	0.227	6 (46.2%)	<0.001
	70–99% (n = 13)	4.0 (0.94)	<0.001	2.3 (1.29)	12 (92.3%)	<0.001	6 (50.0%)	<0.001	12 (92.3%)	<0.001
	Occlusion(n = 14)	4.1 (0.86)	<0.001	2.7 (0.89)	14 (100.0%)	<0.001	10 (71.4%)	<0.001	14 (100.0%)	<0.001

* One patient with symptomatic BAA of 70–99% did not undergo the conventional T1 imaging.

BAA = basilar artery atherosclerosis; BAD = branch atheromatous disease; LAA = large-artery atherosclerosis.

P values were calculated as compared with normal control

T2 plaque signs were present in 46 (61.3%) in the symptomatic BAA patients and 5 (12.2%) in subjects with silent BAA, while none of the normal controls showed T2 plaque sign (Table 2). T2 plaque signs were more frequently found in conjunction with higher degree of basilar artery stenosis (Table 2). In the 46 patients who showed T2 plaque sign, the lateral wall was most frequent location, followed by the dorsal and ventral walls (70.8%, 64.6%, and 33.3%, respectively). In 40 patients with pontine infarction, 29 (76.7%) had lateral wall involvement of T2 plaque signs. T1 hyperintensity was not frequently found in patients with <70% stenosis (Table 2). When considered together with T2 plaque signs, the addition of T1 hyperintensity increased the detection rate of plaque to 16.7% in silent BAA group and 66.7% in symptomatic BAA group (Table 2).

### Diagnostic power of conventional MR markers

The ROC curve showed a high predictive power of maximal BA diameter to distinguish between subjects with any BAA and normal controls (the area under the curve 0.811, 95% CI: 0.745–0.878, p<0.001). The optimal cut-off value was 3.185 mm (sensitivity, 62.9%; specificity, 94.4%). Diagnostic performance of T2 plaque signs alone, T2 plaque signs or T1 hyperintensities, and maximal diameter >3.185mm for predicting BAA of different settings and degrees are shown in Table 3. Overall, increasing sensitivity and decreasing specificity were noted in case of symptomatic BAA and higher degree of stenosis. A maximal diameter >3.185 mm had a higher sensitivity but a lower specificity than T2 plaque signs or T1 hyperintensities as a predictor of BAA.

**Table 3:** Diagnostic Performances of T2 plaque sign alone, T1 hyperintensity or T2 plaque sign, and basilar artery diameter of >3.185mm.

	T2 plaque sign		T1 + T2 (any)		BA diameter >3.185mm	
	Sensitivity	Specificity	Sensitivity	Specificity	Sensitivity	Specificity
Any BAA vs NC	38.8 (29.9–48.3)	100.0 (90.3–100.0)	50.9 (41.3–60.5)	100.0 (90.0–100.0)	62.6 (53.1–71.5)	94.4 (81.3–99.3)
Silent BAA (<30%) vs NC	10.0 (2.8–23.7)	100.0 (90.3–100.0)	16.7 (6.4–32.8)	100.0 (90.0–100.0)	30.8 (17.0–47.6)	94.4 (81.3–99.3)
Symptomatic BAA						
Any degree vs NC	61.3 (61.0–61.7)	100.0 (100.0–100.0)	66.7 (54.7–76.9)	100.0 (87.7–100.0)	78.7 (78.4–79.0)	94.4 (94.2–94.7)
>30% vs <30%	71.2 (70.8–71.6)	92.3 (92.1–92.5)	74.6 (61.3–84.6)	88.2 (75.4–95.1)	84.8 (84.5–85.0)	78.9 (78.5–79.2)
>50% vs <50%	77.5 (77.1–77.9)	78.9 (78.6–79.2)	80.0 (63.9–90.4)	74.3 (62.2–83.7)	87.5 (81.2–87.8)	63.4 (63.0–63.7)
>70% vs <70%	96.3 (96.1–96.5)	76.2 (75.9–76.5)	96.3 (79.1–99.8)	71.1 (59.9–80.3)	88.9 (88.5–89.3)	56.0 (55.6–56.3)

Abbreviations: BA = basilar artery, BAA = basilar artery atherosclerosis, NC = normal control.

### Validation with HRMR

A total of 97 subjects (symptomatic BAA patients, n = 20; subjects with silent BAA, n = 41; normal controls, n = 36) were included in the validation analysis. The presence of atherosclerotic plaque was identified in all patients with symptomatic BAA and subjects with silent BAA. Comparison between the conventional T2WI-based and HRMR-based measurements are shown in Table 4. Patients with symptomatic BAA showed atherosclerotic plaque with a mean diameter of 2.48 (SD 1.39) mm, while silent BAA group had a minimal plaque with a median thickness of 1.22 (SD 0.85) mm. The plaque diameter increased with increasing degree of stenosis (mean 1.18 [SD 0.35] mm in <30% stenosis; mean 1.67 [SD 0.85] mm in 30–50% stenosis; mean 2.02 [SD 1.11] mm in 50–70% stenosis; mean 4.10 [SD 0.73] mm in 70–99% stenosis).

**Table 4:** Comparison between the conventional T2WI-based and HRMR-based measurements.

	Conventional T2WI	HRMR	Difference	P value
Outer diameter (mm)	3.1 (0.87)	3.5 (0.98)	-0.4 (0.49)	<0.001
Plaque diameter (mm)	1.8 (1.15)	2.7 (1.41)	-1.0 (1.16)	0.002

Data are presented as mean (SD).

**Fig 3:**
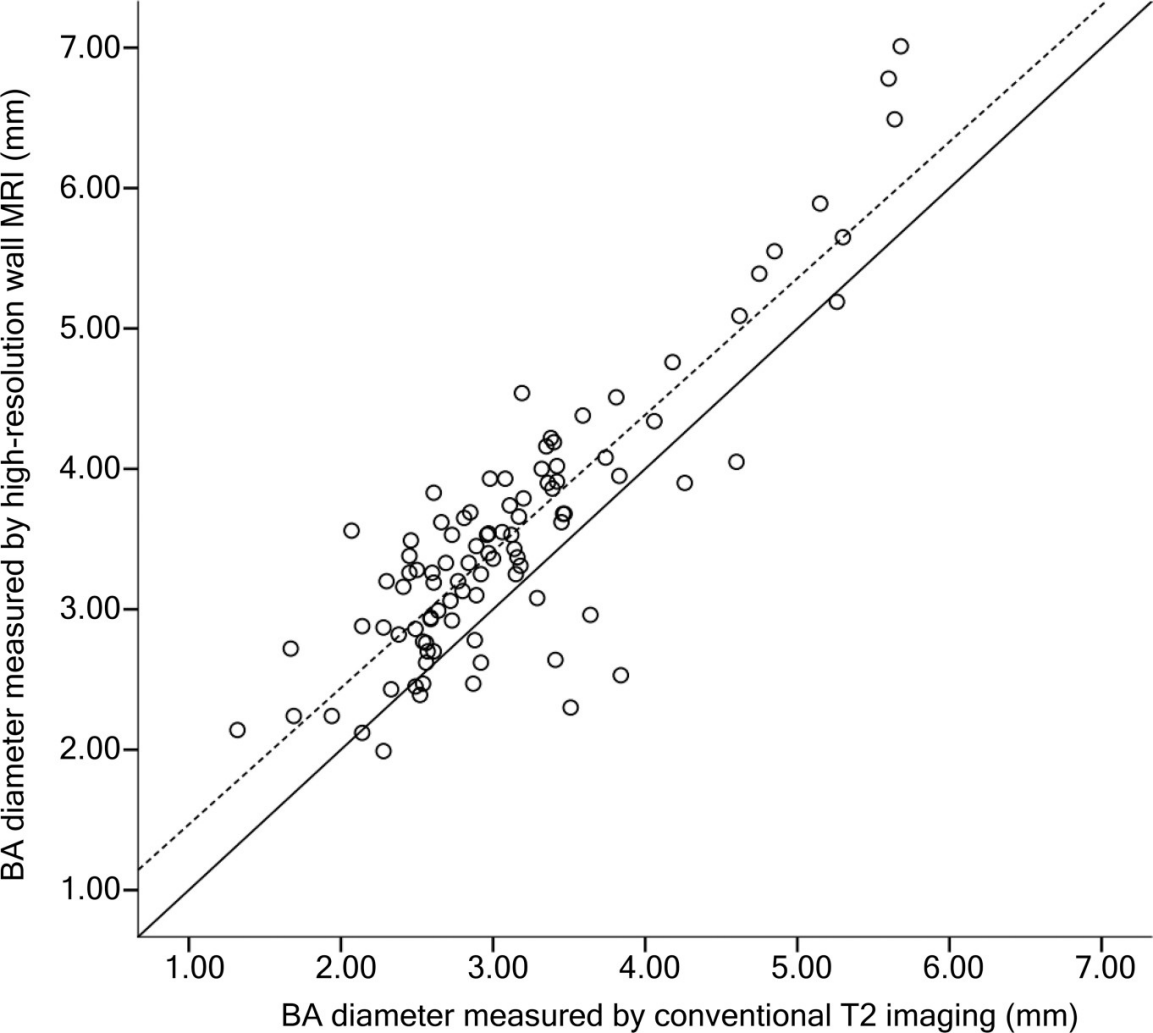
Scatterplot of two basilar artery diameters measured by using conventional T2 MRI (x axis) and high-resolution MR (y axis). The fitted line (dashed line) correlates well with the reference line (black line), where x equals y.

The outer diameter of the BA was mean 4.53 (SD 1.24) mm in patients with symptomatic BAA, 3.43 (SD 0.87) mm in subjects with silent BAA, and 3.12 (SD 0.41) mm in normal controls. The conventional T2-based BA diameter was well correlated with HRMR-based T2 diameter (Pearson’s correlation coefficient = 0.865; Fig 3). Using the linear regression analysis, the regression equation [Ln (BA outer diameter in HRMR) = 0.4253 + (0.2555 × BA diameter by conventional T2-based measurement)] was derived with a good explanatory power (R^2^ = 0.727).

## Discussion

The main findings of the present study were 1) positive remodelling and atherosclerotic plaque of the BA can be documented by conventional T2WI at 3T MRI, 2) both symptomatic and silent BA atherosclerosis can be suspected using markers of conventional MRI, 3) under consideration of its limitation, conventional MRI could be used as an alternative to HRMR where the use of HRMR is limited.

We assessed the role of conventional T2WI at 3T MRI for the suspicion of atherosclerotic plaque. Atherosclerosis is the most common cause of BA steno-occlusive disease [18]. Atherosclerotic plaque can be found even in the absence of relative stenosis [19], and is responsible for progressive motor deficits in pontine infarctions [20]. The course of BA is relatively straight and perpendicular to the MR axis, which enables conventional MRI to visualize the axial cut of the BA. Due to long TR and TE settings, the BA is normally visualized through flow-related signal loss in T2WI [21]. The absence of flow-void has been suggested for the diagnosis of BA occlusion or high-degree stenosis in earlier studies [11,12].

In the present study, T2WI-based measurement of BA diameter provided a clue for BAA, showing a moderate diagnostic performance. Positive remodelling, the compensatory outward expansion of the arterial wall, is an early sign of atherosclerosis [22]. Positive remodelling is more frequent in large artery atherosclerosis than other stroke mechanisms [19]. It has been reported that 63.3% of symptomatic BAA patients showed positive remodelling measured by HRMR [23]. However, HRMR-based measurement of arterial remodelling is prone to underestimation, because the reference vessel is also frequently affected by atherosclerosis. Based on this, findings of positive remodelling might be more common if comparisons were made with normal controls. We found that in some BAA cases, BA diameters were measured at the normal-looking segment but significantly larger than controls, suggesting that atherosclerosis is a diffuse process.

Another finding is that BA plaque can be suspected with conventional T2WI at 3T MRI with a low sensitivity but high specificity. Prior HRMR-based studies have demonstrated the reliability of T2WI for visualizing plaque morphology [24]. We tested the usefulness of conventional T2WI-based plaque identification. Although the sensitivity was low, the specificity was excellent, even in cases with mild stenosis. Furthermore, visualization of the plaque topography may give additional information. In our data, 75% of BAA patients with pontine infarction had involvement of T2 plaque sign in the lateral wall, which gives off the major perforating arteries [25]. This finding warrants future validation study regarding T2WI-based plaque topography and stroke mechanisms.

Intraplaque T1 hyperintensities were present in two-thirds of patients with high degree stenosis, which is comparable with the previous HRMR-based study [7]. Despite the absence of fat-suppression and black-blood technique, our data using conventional T1 hyperintensities showed a high specificity. It might be due to exclusion of central hyperintensities suggesting slow-flow artifact. T1 hyperintensities played a limited but complimentary role to T2WI which has limitation to detect a calcification or intraplaque hemorrhage [26]. Taken together, BA diameter, T2 plaque signs, and T1 hyperintensities may be complimentary to each other. For example, BA diameter can be useful for screening, and T2 plaque signs and intraplaque T1 hyperintensities may be useful as a specific marker.

Our study has some limitations. First, our subjects were from a single center with a relatively small number of patients. Our subjects were Asian, which is ethnic group with the most frequent occurrence of intracranial atherosclerosis. External validation with other ethnicities is warranted. Second, we could not use age-matched controls because of the high prevalence of silent plaques in HRMR of elderly control subjects. However, the BA diameter did not show a correlation with age in the control group, thus age is less likely to serve as a confounder. Third, we judged BA stenosis to be atherosclerosis by means of MR angiogram, which is a method that does not have excellent sensitivity and specificity. However, in all patients who also underwent HRMR, the atherosclerotic plaque was identified. Fourth, the minimal voxel size of 0.469 mm in this study has an in-born measurement error. However, the median difference of BA diameter between patients and controls was more than 1.0 mm, which is greater than the voxel size. Lastly, the sensitivity and specificity of HRMR for detecting intracranial arterial atherosclerosis has not been validated yet. As a surgical specimen of intracranial artery cannot be obtained in living humans, there is no study comparing the intracranial arterial pathology between HRMR and autopsy specimen. However, studies on carotid plaque showed that HRMR well correlated with histopathologic findings [27,28].

In conclusion, increased outer BA diameter and plaque in conventional T2WI at 3T MRI are potentially useful markers of BA atherosclerosis, with a moderate diagnostic performance. T1 hyperintensity may play an additive role. Conventional MR imaging might provide a clue for BA atherosclerosis where the use of HRMR is limited. Future studies should be conducted to correlate conventional MR imaging with HRMR-validated atherosclerotic plaques and to apply them for different stroke mechanisms.

## Supporting information

S1 Fig Examples of T1 hyperintensities. Left column, T2-weighted imaging; Right column, T1-weighted imaging. A-B: Intraluminal T1 hyperintensity related slow flow. C-F: Intraplaque T1 hyperintensity within a T2 plaque sign of mild (C-D) and severe stenoses (E-F)
(TIF)

S1 File Raw dataset. The anonymized dataset used for the investigation is uploaded.
(SAV)
